# Single molecule, full-length transcript sequencing provides insight into the *TPS* gene family in *Paeonia ostii*

**DOI:** 10.7717/peerj.11808

**Published:** 2021-07-15

**Authors:** Jing Sun, Tian Chen, Jun Tao

**Affiliations:** College of Horticulture and Plant Protection, Yangzhou University, Yangzhou, China; Joint International Research Laboratory of Agriculture and Agri-Product Safety, the Ministry of Education of China, Yangzhou University, Yangzhou, China

**Keywords:** *Paeonia ostii*, Single molecule, Full-length transcript sequencing, TPS family members, Functional genes

## Abstract

**Background:**

The tree peony (*Paeonia* section* Moutan* DC), one of the traditional famous flowers with both ornamental and medicinal value, was widely used in China. Surprisingly little is known about the full-length transcriptome sequencing in tree peony, limiting the research on its gene function and molecular mechanism. The trehalose phosphate phosphatase (*TPS*) family genes has been found to affect plant growth and development and the function of *TPS* genes in *Paeonia ostii* is unknown.

**Methods:**

In our study, we performed single molecule, full-length transcript sequencing in *P. ostii*. 10 *TPS* family members were identified from PacBio sequencing for bioinformatics analysis and transcriptional expression analysis.

**Results:**

A total of 230,736 reads of insert (ROI) sequences and 114,215 full-Length non-chimeric reads (FLNC) were obtained for further ORFs and transcription factors prediction, SSR analysis and lncRNA identification. NR, Swissprot, GO, COG, KOG, Pfam and KEGG databases were used to obtain annotation information of transcripts. 10 *TPS* family members were identified with molecular weights between 48.0 to 108.5 kD and isoelectric point between 5.61 to 6.37. Furthermore, we found that *TPS* family members contain conserved TPP or TPS domain. Based on phylogenetic tree analysis, PoTPS1 protein was highly similar to AtTPS1 protein in *Arabidopsis*. Finally, we analyzed the expression levels of all *TPS* genes in *P. ostii* and found *PoTPS5* expressed at the highest level. In conclusion, this study combined the results of the transcriptome to systematically analyze the 10 *TPS* family members, and sets a framework for further research of this important gene family in development of tree peony.

## Introduction

As a traditional ornamental and medicinal flower with a long history of cultivation, tree peony (*Paeonia* section *Moutan* DC) is known as the ‘king of flowers’ in China and belongs to the family Paeoniaceae, symbolizing wealth, prosperity and happiness. Tree peony consists of nine species (Table S1), with *Paeonia ostii* receiving the most extensive promotion at the national level (Ren et al., 2018). Currently, *P. ostii* not only has the characteristics of large flowering, less tillering, high seed yield and strong ecological adaptability, but also is an important new oil crop integrating ornamental, medicinal and oil use (Wang et al., 2019) compared to other nine species. *P. ostii* can be generally found in the Gansu, Anhui, Shanxi and Henan provinces of China. It is a diploid cultivated tree peony species, with 2n =2x =10 chromosomes, clearly characterized by white or pale rose colored petals without a base blotch (Hong, Pan & Pan, 2004). At present, research on tree peony has mainly focused on its morphological characteristics, cytological characteristics and germplasm resources. Surprisingly, full-length transcriptome sequencing in tree peony has not been performed.

The trehalose phosphate phosphatase (*TPS*) gene family in higher plants is a small gene family that is divided into two distinct classes, class I and class II (Lunn, 2007). Obviously, they are different in copy number, gene expression patterns, enzyme activity and physiological functions (Ping et al., 2019). Currently, in *Arabidopsis* only class I *TPS* genes have been reproducibly shown to encode catalytically active TPS enzymes (Blazquez et al., 1998; Van Dijck et al., 2002; Vandesteene et al., 2010), and the functions of noncatalytic class II TPS-like proteins are poorly understood (Harthill et al., 2006; Lunn et al., 2014). Although significant progress has been made in the study of *TPS* genes, a number of *TPS* gene families have been identified in only some model plants. For example, there are 11 *TPS* members in *Arabidopsis* (*AtTPS1-11*) (Shima et al., 2007) and in rice (*OsTPS1-11*) (Vandesteene et al., 2010), while 12 members were found in poplar (*PtTPS1-12*) (Tuskan et al., 2006; Vandesteene et al., 2010).

The *TPS* gene family also shows widespread functional diversification. Catalyzed by TPS, the biosynthetic precursor of trehalose, trehalose-6-phosphate (T6P) plays a crucial role, participating in the physiological processes of plant embryo development, flower induction, senescence regulation and seed filling, and in the respond to biotic and abiotic stresses (Gomez et al., 2010; Kumar et al., 2019; Wahl et al., 2013; Wingler et al., 2012; Zhao et al., 2019). For example, overexpressing *OsTPS1* in rice enhanced its tolerance to salt, drought and low temperatures (Jang et al., 2003). Moreover, *SlTPS1* in the *TPS* gene family of *Selaginella lepidophylla* participates in the response to heat and salinity by enhancing T6P biosynthesis (Zentella et al., 1999). When *AtTPS1* was deleted, it caused embryo arrest, hindered vegetative growth and delayed flowering of *Arabidopsis thaliana* (Eastmond et al., 2002; Gomez et al., 2006; Gomez et al., 2010; Van Dijken, Schluepmann & Smeekens, 2004; Wahl et al., 2013). The product of the TPS reaction, T6P, functions as a signal for sucrose availability (Yadav et al., 2014) and it is involved in triggering axillary bud outgrowth in garden pea (*Pisum sativum* L) (Fichtner et al., 2017). In potato tubers, T6P is a pivotal mechanism linking growth, development and metabolism by SnRK1 signaling (Debast et al., 2011). Similar studies also suggested that when T6P is deleted, the size of the pea seeds will decrease, and the starch yield will decrease because auxin acts downstream of T6P to facilitate seed filling (Meitzel et al., 2019). However, whether the members of *TPS* gene family have an effect on the development of *P. ostii* and their underlying molecular mechanisms remains unknown.

Transcriptome research is one of the essential tools to understand the life process. However, most of the previous technologies are not capable of generating reads representing entire transcripts due to sequencing read shortness. The second-generation high-throughput sequencing platforms, such as Illumina sequencing platforms often cannot obtain or assemble the complete transcript entirely and recognize isoforms, homologous genes, and the transcripts of superfamily genes and alleles, making it challenging to accurately detect transcript isoforms generated by alternative splicing (AS) events (Bessa et al., 2020) and structural variations (SVs) (Sharon et al., 2013; Wang et al., 2016b). Moreover, NGS has difficulty in distinguishing among different transcripts possessing identical exons (Tilgner et al., 2013) and has the disadvantage of high GC preference in analyzing the original sequencing reads.

Currently, the outcomes of third-generation sequencing, single-molecule, real-time (SMRT) sequencing developed by Pacific Biosciences has enabled the generation of kilobase-sized sequencing reads (Gordon et al., 2014), showing more RNA molecules (Sharon et al., 2013). The RACE technique requires no interruption of the RNA fragments or reverse transcription of the full-length cDNA (Thomas et al., 2014). By employing appropriate methods for cDNA preparation, bona fide full-length transcript sequences can be generated. SMRT sequencing reads exhibit high sequencing error rates, most notably base insertions or deletions. However, due to the random nature of the encountered errors, the construction of highly accurate consensus by reiterated sequencing of the same fragment is straightforward. Currently, SMRT sequencing has been applied to investigate the full-length transcriptome in diverse species, such as wheat (Dong et al., 2015), *Salvia miltiorrhiza* (Xu et al., 2015), sorghum (Abdel-Ghany et al., 2016), maize (Wang et al., 2016a), sugarcane (Hoang et al., 2017b), perennial ryegrass (Xie et al., 2020), *Trifolium pratense* L. (Chao et al., 2018), Chinese cabbage (Tan et al., 2019) and *Crocus sativus* L (Qian et al., 2019).

The objective of this study was to apply PacBio full-length sequencing to provide a new reference gene database for the further study of *P. ostii*. In this paper, young leaves, roots, stems, seeds, flowers and fruit pods were collected for Single molecule, full-length transcript sequencing to analyze the *P. ostii* transcriptome. Then, we identified the members of the *TPS* gene family, providing valuable gene resources for further research into the evolution and biological functions in *P. ostii*.

## Materials & Methods

### Plant materials and sample preparation

**Figure 1:**
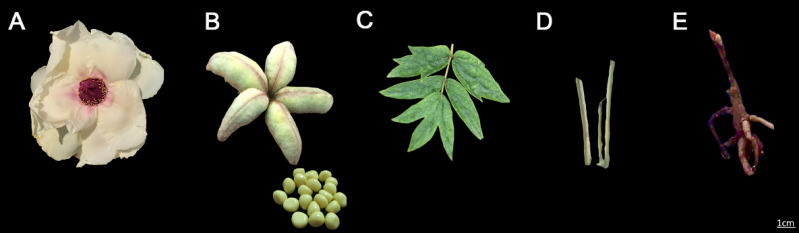
The tissues of *Paeonia ostii* used in this study. (A) The flower in full bloom. (B) Pods and seeds. (C) Leaves. (D) Stems. (E) Roots.

Young leaves, stems, roots, flowers, seeds and fruit pods of three different 3-year-old *P. ostii* from the germplasm repository of the Horticulture and Plant Protection College, Yangzhou University, Jiangsu Province, P.R. China (32°23′31′N,119°24′50′E) were collected and stored in liquid nitrogen for RNA extraction. They had been grown under the same conditions for three years. Seeds, leaves and stems used for qRT-PCR were collected from the same strain of three different *P. ostii* at the developmental stages of 30, 50 and 70 days after flowering (DAF) in 2019, because the endosperm of seeds begins to develop after 30 DAF and becomes cellular after 70 DAF (Yao, 2020). Leaves, stems, roots, and flowers in the bloom period of *P. ostii* were collected in April 2019. Fruit pods and seeds were collected at 70 DAF in June 2019. RNA was extracted separately from leaf, stem, root, flower, seed and pod tissues (Figs. 1A–1E) using the CTAB method. To ensure the accuracy of the sequencing data, all RNA samples were checked using a Nanodrop 2000C (Thermo Scientific). The RNA integrity was checked using an Agilent 2100 bioanalyzer (Agilent Technologies), which included RIN, 28S/18S and 5S peaks. Electrophoresis was used to detect whether the RNA samples contained gDNA contamination and assess RNA quality by identifying the ribosomal bands. gDNA were digested by using the Exonuclease I (NEB, M0293S).

### PacBio library construction and sequencing

After RNA quality inspection, we first mixed equal quantities of high-quality RNA from different organs of one *P. ostii*. Then, the mixed samples from three different *P. ostii* were mixed as one pool (Fig. S1). Ten micrograms of RNA from the sample pool was manipulated as follows: (1) reverse transcribed into cDNA using the SMARTer PCR cDNA Synthesis Kit to prepare full-length, high-quality cDNAs with VN Primer (2 µM Nanopore) and Strand-Switching Primer (10 µM Nanopore) (Table S2); (2) a BluePippin™ Size Selection System (Sage Science, Beverly, MA) was used to screen full-length cDNA fragments and construct cDNA libraries of different sizes (1–2 kb, 2–3 kb, and 3–6 kb); (3) the amplified full-length cDNA was reamplified by PCR and end-repair of the full-length cDNA; (4) then, each SMRT bell library (500 ng size-selected cDNA) was constructed using the Pacific Biosciences DNA Template Prep Kit 2.0; (5) after digestion by Exonuclease I (NEB, M0293), secondary screening was carried out with BluePippin to obtain a sequencing library. After the completion of the library construction, the quality of the library was tested: Qubit 2.0 was use for accurate quantification. The library size was tested using the Agilent 2100, and the library size was as expected before on-board sequencing (the concentration of Qubit >2 ng/µL and the total amount >50 ng/µL). After the library was purified by AMpure PB Beads, full-length transcriptome sequencing was performed using PacBio RSII according to the target data volume.

### Full-length corrected transcript collection in SMART sequencing

Full-length transcriptome sequencing based on PacBio SMRT (Sequencing By Synthesis, SMRT) single-molecule real-time sequencing technology does not require the disruption of RNA fragments, and the full-length cDNA had been obtained by reverse transcription using RACE technology. The long read (median 10 kb) of the platform contains a single complete transcript sequence information without post assembly analysis (Gordon et al., 2015; Thomas et al., 2014). The analysis process for acquiring the full-length transcriptome mainly includes three stages (Sharon et al., 2014): (1) Full-length sequence identification. Convert all original sequences to ROI (Reads Of Insert) sequences based on the adaptor. According to whether there are 3′ primers, 5′ primers, or Poly A (optional), they are divided into two categories: full-length and non-full-length sequences. (2) Isoform level clustering to obtain consensus sequences. ROI sequences from the same transcript were clustered using the ICE (Iteratively Clustering and Error Correction) algorithm, and each cluster obtained a consensus sequence. (3) Consistent sequence polishing. The non-full-length sequence was used to perform the polishing on the obtained consensus sequence to obtain a high-quality sequence for subsequent analysis. The resulting transcript sequences can be directly used for subsequent isoform, homologous gene, gene family, SSR, alternative splicing, lncRNA and other analyses.

The PacBio RS II used for SMRT sequencing contained 150,000 Zero-Mode Waveguides (ZMWs) per cell. The reads were sequenced into ZMW wells, and one ZMW contained reads as valid data. The ROI sequences satisfying the conditions of full passes ≥0 and accuracy ≥0.75 were extracted. To obtain the iteratively clustered sequences, the iterative clustering for error correction algorithm was performed by the SMRT Analysis (v2.3.0). Combined with a non-full-length sequence, high-quality transcripts (HQs) with accuracies greater than 99% were obtained. In the process of full-length transcript sequencing, the 3′ end has a poly-A structure, and it can be determined that the 3′ end is relatively intact, and the 5′ end sequence may be degraded, resulting in different copies of the same transcript being assigned to different clusters. CD-HIT (Li & Godzik, 2006) software was used to combine sequences with high similarity and to remove redundant sequences from among the high-quality transcripts.

### Functional annotation, ORFs, SSR analysis and lncRNA identification

We used BLAST to combine the obtained nonredundant transcript sequences with the NR (http://www.ncbi.nlm.nih.gov) (April, 2021), SwissProt (http://www.ebi.ac.uk/swissprot) (April, 2021), GO (http://www.geneontology.org) (April, 2021), COG (http://www.ncbi.nlm.nih.gov/COG) (March, 2021), KOG (https://mycocosm.jgi.doe.gov/help/kogbrowser.jsf) (April, 2021), Pfam (http://pfam.xfam.org/) (March, 2021) and KEGG (http://www.genome.jp/kegg) (April, 2021) databases to obtain annotation information of the transcripts (Altschul, 1999).

TransDecoder software 2.0.1 (https://transdecoder.github.io/) is based on the length of the open reading frame (ORF), log-likelihood score and the comparison of amino acid sequences and protein domain sequences in the Pfam database, which can identify reliable coding sequences (CDSs) from the transcript sequence.

Simple repetitive sequences were identified through MISA software. Seven types of SSRs were identified by analyzing the sequences of the transcripts: mononucleotide, dinucleotide, trinucleotide, tetranucleotide, pentanucleotide, hexanucleotide and compound SSRs.

We filtered out the transcripts with coding potential to obtain the predicted lncRNAs. In this study, the most widely used coding potential analysis method was used to predict the lncRNAs among the transcripts, including four methods: CPC analysis (Kong et al., 2007), CNCI analysis, Pfam protein domain analysis, and CPAT analysis.

### Quantitative Real-Time PCR anlaysis

Each organ was represented by three biological replicates with three technical replicates. Total RNA from the seeds, leaves and stems from three *P. ostii* was extracted by a MiniBEST Plant RNA Extraction Kit (TaKaRa, Japan). Then, the cDNA was synthesized by the PrimeScript RT reagent Kit with gDNA Eraser (TaKaRa, Japan) (Zhao et al., 2020). Before qRT-PCR, we used water instead of cDNA and ran the PCR. The agarose gel electrophoresis showed clear objective band, so there was no pollution in the PCR system. A BIO-RAD CFX Connect Optics Module (Bio-Rad, Des Plaines, IL, U.S.A.) was used to analyze the *PoTPS* expression levels. Expression values were calculated by the 2^−ΔΔ*Ct*^ comparative threshold cycle (C_t_) method (Livak & Schmittgen, 2001). SYBR Premix Ex Taq (Perfect Real Time) (TaKaRa, Japan) with 12.5 µL 2 × SYBR Premix Ex Taq, 2 µL cDNA solution, 8.5 µL ddH_2_O, and 2 µL solution of mix primers were the system to perform qRT-PCR. Amplification conditions: 95 °C for 30 s, 40 cycles at 95 °C for 5 s, 52 °C for 30 s, and 72 °C for 30 s. The *ubiquitin* gene (JN699053) was used as an internal reference for this experiment and the expression level of this reference gene was stable in all organs of *Paeonia suffruticosa* (Wang et al., 2012). All primers were listed in Table S2.

### Analysis of the *TPS* genes family in *Paeonia ostii*

To classify the *PoTPS* genes in *P. ostii*, Cluster X 2.0.12 software (http://www.cluster-x.org/) was applied for multiple sequence alignment by using protein sequences of *Arabidopsis*. To construct the phylogenetic tree, neighbor-joining (NJ) method was used by MEGA 7.0 software, and bootstrap values were set as 1000 bootstrap replicates (Saitou & Nei, 1987). The conserved motifs of the *PoTPS* sequences were identified by the MEME program (https://meme-suite.org/meme/), and the parameters were set as a maximum of 10 motifs and an optimum motif width of 6-200 amino acid residues (Zhao et al., 2018). The conserved domains were visualized using the TBtools software.

## Results

### *Paeonia ostii* transcriptome sequencing with SMRT

**Figure 2:**
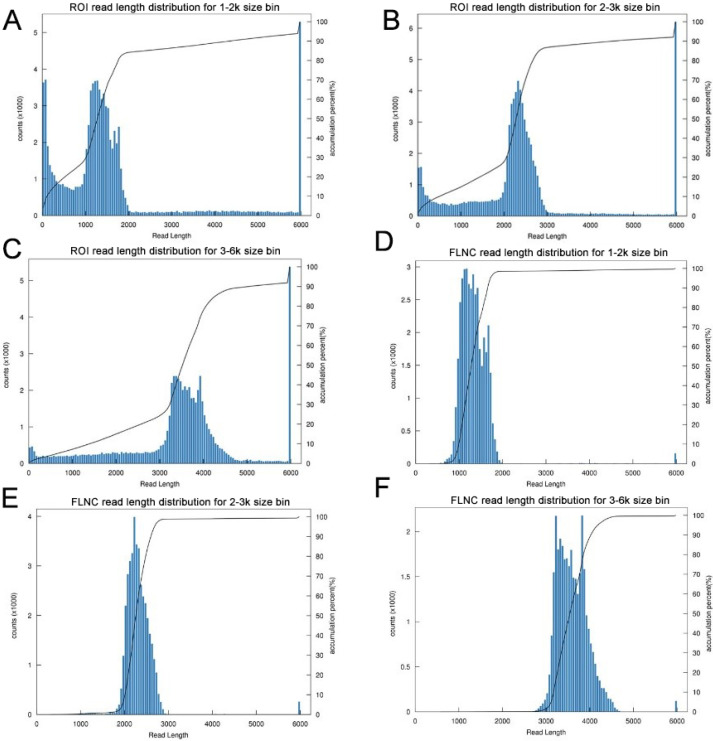
Summary of PacBio SMRT ROI and FLNC sequences. (A–C) Number and length distributions of 230,736 ROI sequences from 1–2 k, 2–3 k and 3–6 k sized libraries. (D–F) Number and length distributions of 114,908 FLNC reads from 1–2 k, 2–3 k and 3–6 k sized libraries.

**Figure 3:**
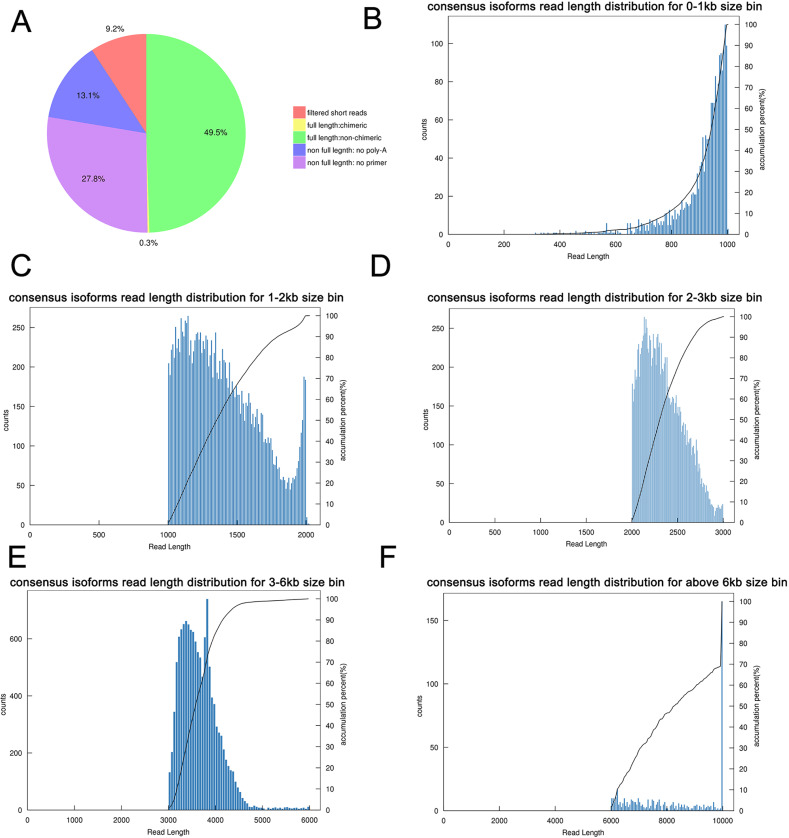
Summary of PacBio SMRT sequencing. (A) Proportion of different types of PacBio reads. (B–F) Number and length distributions of 45,006 consensus isoforms reads from <1 kb, 1–2 kb, 2–3 kb, 3–6 kb and >6 kb.

The transcriptome of the pooled samples was sequenced and analyzed with the PacBio Sequel platform to accurately capture the full-length sequences. A total of three libraries divided by cDNA lengths of 1–2 kb, 2–3 kb and 3–6 kb were constructed. Then, 3,568,378 subreads (8.49 Gb) and 343,264 polymerase reads were obtained after filtering. These data have been deposited in the National Center for Biotechnology Information (NCBI) (BioProject accession: PRJNA688625). The ROI sequence was extracted from the original sequence according to the condition that full passes ≥0 and the sequence accuracy ≥0.75. To evaluate the offline date, the number of ROIs, the number of bases of the ROIs and the mean length of the inserted sequence in each library were counted. In total, we obtained 230,736 ROI sequences and the mean read quality of the insert of each size-selected library was 0.92 (Table S3). The ROI read length distribution of each size-selected library had three consistent peaks (Figs. 2A–2C). By filtering out short fragments <300 bp, the sequences containing both the 3′primers and 5′ primers at the same time and having a poly A tail before the 3′ primers were defined as full-length sequences. After further filtering and analysis, 114,215 full-length nonchimeric (FLNC) reads were obtained. The FLNC read length distribution of each size-selected library is shown in Figs. 2D–2F. The trends of the three peaks were consistent, which was in line with the expectations. The percentage of FLNC reads in all ROI sequences was 49.5% (Fig. 3A), accounting for half of the number of all ROI sequences, combined with the ROI and FLNC sequences, which showed that the quality of the transcriptome was better.

**Figure 4:**
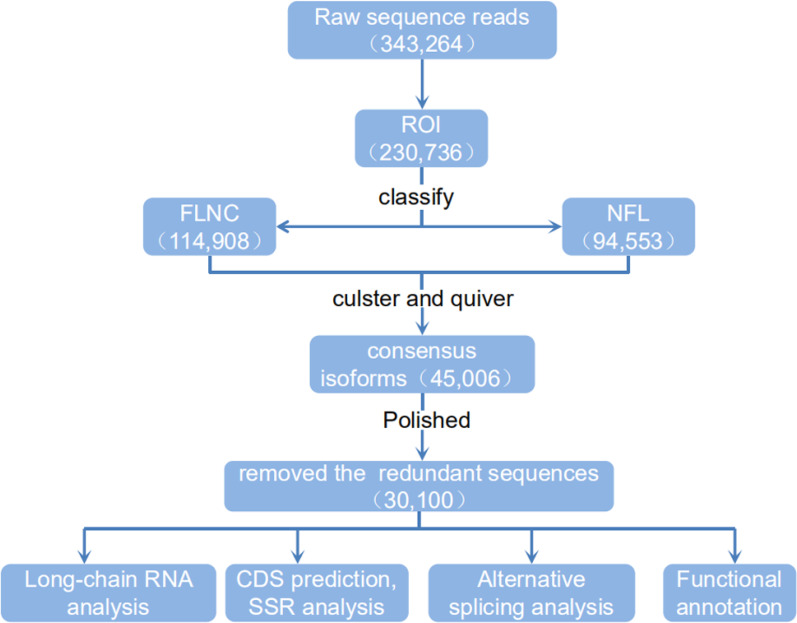
Flow chart of bioinformation analysis. The raw sequence reads from a PacBio Rs sequencer were sorted into full and non-full length reads using a classification algorithm that identified full length reads with forward and reverse primers, as well as a poly A tail. Iterative clustering for isoforms (ICE) was performed on full length reads, and non-full-length reads were recruited to perform ARROW polished on the consensus isoforms. Polished sorted reads into high and low quality bins, and either high quality data, all sequence data or both sets of data, were carried on to further applications.

By using IsoSeq of SMRT Analysis software to perform cluster analysis on the full-length sequence and combining the non-full-length sequences, 45,006 identical transcripts were obtained (Fig. 4). The consensus isoform read length distribution of each size bin (<1, 1–2, 2–3, 3–6 and >6 kb) is shown in Figs. 3B–3F, and the average consensus isoform read lengths were 912, 1399, 2346, 3665 and 9297 (Table S4). After correcting the consistent sequences in each cluster by quiver program, only 8,239 low-quality isoforms (LQs) and 36,767 high-quality isoforms (HQs) were obtained. Among them, 0–1 kb high-quality transcripts accounted for the largest proportion (91.58%) of consensus isoform reads (Table S4), indicating that the shorter the cDNA length, the more high-quality reads were obtained. Finally, by using CD-HIT to remove the redundant sequences of the high-quality sequences, 30,100 transcript sequences were obtained for subsequent analysis (Fig. 4).

### ORF and transcription factor prediction, SSR analysis and lncRNA identification

**Figure 5:**
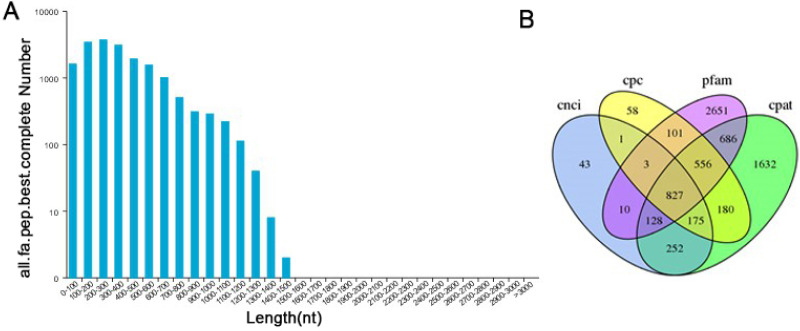
Information on the distribution of ORFs and lncRNAs. (A) The length distribution of 17,904 completed ORFs. (B) Venn diagram of lncRNAs predicted by CPC, CNCI, CPAT and Pfam methods.

TransDecoder software was used to predict the coding protein sequences and to identify full-length ORFs. In our study, a total of 28,415 ORFs were obtained, of which 17,904 were complete ORFs. Sequences with start and stop codons were defined as complete coding sequences. The predicted length distribution of the complete ORFs is as follows (Fig. 5A). Transcripts >500 bp in length were selected. By using MISA software to analyze 30,086 transcripts, 9,789 SSR- containing sequences from among the 13,632 evaluated SSRs were identified. Large numbers of SSR containing sequences were mono-, di-, or trinucleotide repeats (Table S5). Currently, long noncoding RNAs (lncRNAs) play an important role in many life activities, such as dose compensation effects, epigenetic regulation, cell cycle regulation and cell differentiation regulation, and they have become a hotspot of genetic research. Therefore, it is very meaningful to analyze lncRNAs through transcriptome analysis. Four methods were used to make lncRNA predictions for the transcripts CPC, CNCI, Pfam and CPAT. To visually display the analysis results, the noncoding transcripts identified by the above four analysis methods were taken into intersection for subsequent lncRNA analysis (Fig. 5B). A total of 827 lncRNAs were predicted by filtering out the transcripts <300 bp in length.

### Functional annotation of the transcripts

**Figure 6:**
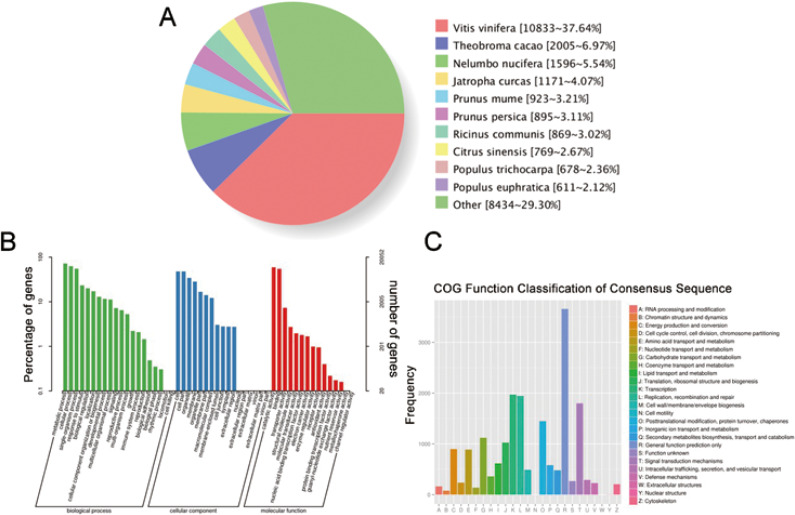
Function annotation of transcripts. (A) Nr Homologous species distribution. (B) Distribution of GO terms for all annotated transcripts in biological process, cellular component and molecular function. (C) COG function classification of consensus sequence.

We have submitted the final polished consensus mRNA sequences to NCBI (accession number GJFW00000000). Blast software was used to compare the nonredundant transcripts with the NR, SwissProt, GO, COG, KOG, Pfam, and KEGG databases. A total of 28,850 transcript annotation information points were obtained (Table S6). We searched for homologous species through sequence alignment. The 28,790 transcripts in Nr were aligned as shown below, showing that the largest number of transcripts were distributed in *Vitis vinifera* (37.64%) (Fig. 6A). The GO annotation system includes three main branches, namely biological process, molecular function and cellular component. Among them, biological processes involved metabolism, cellular and single-organism processes, responses to stimulus and biological regulation. Cell components mainly involved cells, cell parts, organelles, membranes and other parts. Molecular functions involve catalysis, binding, transport, transcription activities and other activities. The statistical results of 20,052 transcripts of GO are shown in Fig. 6B. The COG (Cluster of Orthologous Groups of proteins) database was constructed based on the phylogenetic relationships of the bacteria, algae and eukaryotes. The COG database can be used to classify gene products as orthologues. The COG classification statistics of 12,892 transcripts are shown in Fig. 6C.

### Identification of the *TPS* gene family

**Figure 7:**
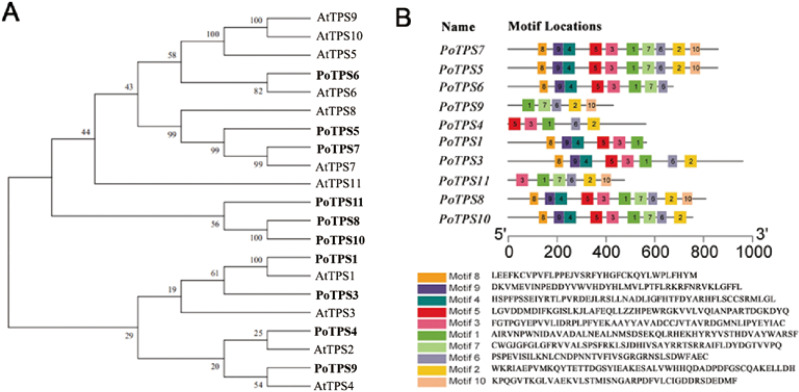
Bioinformatics analysis of *PoTPSs* members. (A) Phylogenetic relationships of the TPS proteins between *Paeonia ostii* and *Arabidopsis*. Bootstrap values only above 60 are provided above branches. (B) Schematic diagram of amino acid motifs of PoTPS protein. The TPS domain includes 1, 3, 4, 5, 8, 9 modifs and TPP domain includes 2, 6, 7, 10 modifs.

Ten *TPS* family members were selected and identified from the transcriptome sequencing with the SMRT database for *P. ostii*, and the accession numbers for the 10 *TPS* genes are MW700299–MW700308. According to the percentage of similarity between these *TPS* family members and *AtTPS1-11* in *Arabidopsis*, we named these genes *PoTPS1*, *PoTPS3*-*PoTPS11* (Table S6). To clarify the related protein information of the TPS family in *P. ostii*, ProtParam was used to analyze the physical and chemical properties and secondary structure elements of the TPS family members in this study. The number of amino acids was approximately 429-959, the isoelectric point ranged between 5.61−6.37 and the protein molecular weight was between 48.0–108.5 kD. The secondary structure is mainly composed of a helix, extended strand and random coil. Specifically, the random coil structure of 10 TPS members was above 29%, which is beneficial to maintaining the stability of TPS proteins (Table 1). Using Pfam and SMART to analyze protein domains, it was found that these TPS proteins all have conserved domains, which contain two typical characteristics of TPS (Pfam: Glyco-transf-20) and TPP (Pfam: Trehalose-PPase) structural domains. In particular, *PoTPS1* contains only one TPS domain, and the remaining nine TPS family members all contain one TPS domain and one TPP domain. In addition, it was also found that the distribution of the domains of the TPS family members is roughly the same as that of *Arabidopsis*, rice and soybean, showing that the TPS and TPP domains of this family are positionally conserved among different species. However, whether these family members are functionally similar is not yet known. To analyze the genetic relationship and evolution characteristics of the *TPS* members in *P. ostii*, a phylogenetic tree was constructed by using MEGA 7.0 software, finding that the PoTPS1 protein has a high similarity to AtTPS1 and that the PoTPS7 protein has a high similarity to AtTPS7 (Fig. 7). In addition, the motif is a small conserved sequence fragment. In biology, a mathematical statistical model based on data can help predict the reliability of phylogenetic analysis, so MEME was used to analyze the motifs of all TPS proteins and it was found that only PoTPS5, PoTPS7 and PoTPS8 had ten conserved motifs, while the other proteins had 5-9 motifs each (Fig. 7).

**Table 1:** The physical and chemical properties and secondary structure elements of TPS family members.

**Gene name**	**Number of amino acids**	**Molecular weight (kD)**	**Isoelectric point (pl)**	**Aliphatic index**	**Protein hydrophobicity**	**Helix (%)**	**Extended strand (%)**	**Random coil (%)**
*PoTPS1*	565	64.17	6.33	89.20	−0.291	51.50	13.81	29.73
*PoTPS3*	959	108.46	5.77	85.29	−0.337	41.40	14.08	39.10
*PoTPS4*	561	62.63	5.81	89.66	−0.249	42.42	14.44	36.90
*PoTPS5*	856	97.06	5.61	90.95	−0.214	43.81	16.94	34.11
*PoTPS6*	674	76.82	6.37	94.12	−0.138	45.10	16.62	31.75
*PoTPS7*	858	96.65	6.32	85.77	−0.227	42.66	16.90	35.78
*PoTPS8*	807	91.49	6.19	89.63	−0.223	44.24	16.23	34.70
*PoTPS9*	429	48.01	6.15	85.38	−0.206	44.76	17.02	33.33
*PoTPS10*	754	86.10	6.16	90.49	−0.224	44.69	16.84	33.16
*PoTPS11*	474	53.09	5.85	84.56	−0.299	45.36	16.24	33.76

#### Gene expression analysis

**Figure 8:**
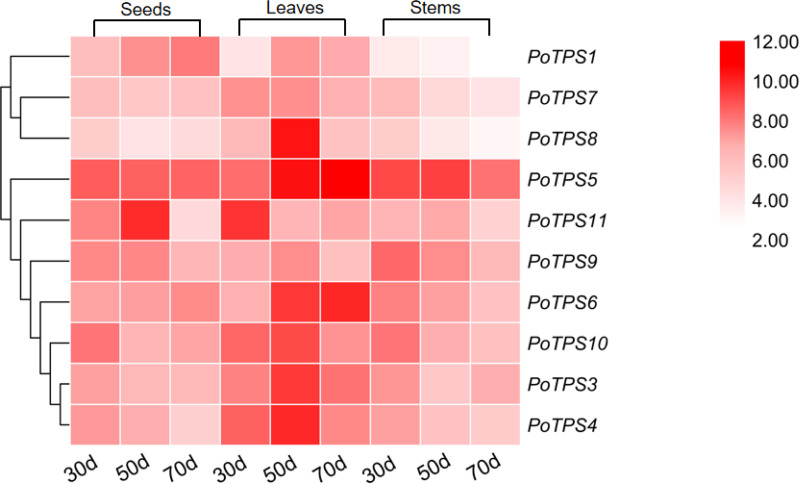
Expression profiles of the 10 *PoTPS* genes in different tissues and different periods. Seeds, leaves and stems of 30d, 50d, 70d after flowering were used to analyze the expression levels of 10 *PoTPS* genes in *Paeonia ostii*. The red from light to dark represents the expression level from low to high.

We analyzed the expression levels of 10 *TPS* family members on the stems, leaves and seeds at 30, 50, and 70 DAF to explore whether the *TPS* gene expression in different tissues and different periods follows certain expression patterns and whether *TPS* genes were specifically expressed in different tissues. TBtools software was used to analyze the obtained PCR results and the red color from light to dark represents the expression level from low to high. The results showed that in seeds the relative expression levels of *PoTPS5* and *PoTPS6* were relatively high at 30 DAF, and the relative expression levels of *PoTPS5* and *PoTPS11* were high at 50 DAF. The relative expression levels of *PoTPS1* and *PoTPS5* were relatively high at 70 DAF. Obviously, the relative expression level of *PoTPS1* in seeds tended to increase with the development of the seeds. In leaves, the expression of *PoTPS11* was higher at 30 DAF, the expression of *PoTPS5* and *PoTPS8* was higher at 50 DAF, and the expression of *PoTPS5* was highest at 70 DAF. In stems, the relative expression level of *PoTPS5* was high regardless of the period (Fig. 8). In summary, the relative expression of *PoTPS5* was always been at a high level in different tissues at different periods.

## Discussion

Tree peony, as a traditional Chinese flower, is worthy of research by gardeners and landscape researchers to improve its breeding. The current understanding of the tree peony transcriptome is mainly devoted only to the transcriptome of specific tissues such as petals (Li et al., 2017). Surprisingly, little full-length transcriptome sequencing has been conducted for tree peony. In our study, SMRT-seq allowed us to obtain information about the full-length transcriptome sequence of tree peony. We obtained 230,736 ROI sequences and 114,215 FLNC transcripts for further ORF and transcription factor prediction, SSR analysis and lncRNA identification. We circumvented the shortcomings of short reads in RNA-Seq2.0 and obtained long reads that can represent the entire transcript, confirming that SMRT-seq was more accurate in recovering full-length transcripts (Hoang et al., 2017a). Since the complete peony genome has not yet been disclosed, the massive amount of full-length transcripts obtained in this study provide a reliable biological information background resource for the subsequent in-depth study of the molecular mechanism of peony growth and development. It has laid an important foundation for molecular breeding.

*TPS* family members in *Arabidopsis* (Vandesteene et al., 2010), poplar (Yang et al., 2012), rice (Zang et al., 2011), cotton (Xie et al., 2015), lotus (Jin et al., 2016), potato (Xu et al., 2017) and apple (Du et al., 2017) have been genome-wide identified. Based on the transcriptome result, a total of 10 *TPS* family members in *P. ostii* were identified. Except for PoTPS1 which only contains the TPS domain, other PoTPS proteins contain both conserved TPS and TPP domains, similar to those in *Arabidopsis*, poplar, rice and drumstick trees (Lin et al., 2018; Yang et al., 2012). The TPS and TPP domain of this family are positionally conserved across different species. Studies have shown that genes lacking a TPP conserved domain in the C-terminus in *Arabidopsi* s can cause catastrophic consequences for the plant (Fichtner et al., 2020). Through anlaysis of the phylogenetic tree, it was found that the PoTPS1 protein is very similar to AtTPS1 (Fig. 7). AtTPS1 is a key enzyme for Tre6P synthesis in *Arabidopsis* and is essential for embryogenesis and normal postembryonic growth and development (Fichtner et al., 2020). Therefore, whether the deletion of the TPP domain of *PoTPS1* has a good or bad effect on the growth and development of *P. ostii* is not yet known.

The expression levels of *PoTPS* genes are totally different in seeds, stems and leaves at different periods. Surprisingly, *PoTPS5* showed the highest transcript level in different organs at different periods (Fig. 8). In *Arabidopsis*, *TPS5* is involved in affecting the trehalose content and functions as a negative regulator of ABA signaling (Tian et al., 2019). Whether the high expression of *PoTPS5* represents a positive effect on the growth of *P.ostii* needs further study. Moreover, it is worth noting that expression levels of *PoTPS1* increase during seed development (Fig. 8). Based on this qRT-PCR result, we can predict the pathway of *PoTPS1* to participate in the biosynthesis and accumulation of trehalose in seeds. When T6P synthesized by TPS is lacking, the size of pea seeds will decrease, and the starch yield will decrease because auxin acts downstream of T6P to facilitate seed filling (Meitzel et al., 2019). It can also be hypothesized that *PoTPS1* may be involved in the development and filling process of seeds in *P. ostii*. The cell-specific pattern of TPS expression remains to be examined. Fortunately, previous studies have found that *AtTPS1* is normally expressed in reproductive organs, roots, leaves of all major tissues (Schmid et al., 2005). For example, it was shown that the CsTPS1 protein is contained in phloem sap exudates in *Cucumis sativus* (Hu et al., 2016). In contrast, in *Arabidopsis*, the number of *AtTPS1* transcripts is quite large as shown by ribosome pull-down experiments in bundle sheath cells (Aubry et al., 2014). Therefore, to better explore the specific functions of the *PoTPSs*, it is worthwhile to study the relationship between *PoTPSs* and biomass accumulation in different tissues in future studies.

## Conclusions

In this study, we analyzed the full-length transcriptome of *P. ostii* with SMRT-seq technology. Our findings provide valid information for improving tree peony draft genome annotation and full characterization of its transcriptome. Moreover, we identified 10 TPS family members in *P. ostii* and provided a comprehensive analysis of their physical and chemical properties, conserved protein motifs and phylogenetic tree analysis. Finally, we analyzed the expression patterns of *PoTPSs*, helping us to better understand the functions of these *TPS* family members.

##  Supplemental Information

10.7717/peerj.11808/supp-1 Supplemental Information 1 The nine species of tree peony

10.7717/peerj.11808/supp-2 Supplemental Information 2 Primers used for reverse transcription and qRT-PCR

10.7717/peerj.11808/supp-3 Supplemental Information 3 Summary of ROIs from PacBio SMRT

10.7717/peerj.11808/supp-4 Supplemental Information 4 Raw qRT-PCR data for Fig. 8

10.7717/peerj.11808/supp-5 Supplemental Information 5 MW700299–MW700308

10.7717/peerj.11808/supp-6 Supplemental Information 6 Summary of consensus sequence from PacBio single-molecule long-read sequencing

10.7717/peerj.11808/supp-7 Supplemental Information 7 Annotated databases New Isoform Number

10.7717/peerj.11808/supp-8 Supplemental Information 8 Melting curves

10.7717/peerj.11808/supp-9 Supplemental Information 9 Prediction of SSRs out of our transcript datasets

10.7717/peerj.11808/supp-10 Supplemental Information 10 The percentage of similarity between these *TPS* family members and *AtTPS1-11* in *Arabidopsis*

10.7717/peerj.11808/supp-11 Supplemental Information 11 The flow chart of sample preparation

10.7717/peerj.11808/supp-12 Supplemental Information 12 All Database annotation

## Additional Information and Declarations

### Data Availability

The following information was supplied regarding data availability: The PCR data and annotations regarding GO, KEGG are available in the Supplemental Files. The data are available at the Sequence Read Archive (SRA): PRJNA688625 and the 10 PoTPS genes are available at NCBI: MW700299–MW700308. The final polished consensus mRNA sequences are available at NCBI: GJFW00000000.
