# Supplementary material for: Single molecule, full-length transcript sequencing provides insight into the TPS gene family in Paeonia ostii
Source: PeerJ. 2021 Jul 15;9:e11808. doi: 10.7717/peerj.11808 (PMC8286706; doi:10.7717/peerj.11808)
Supplement: Supplemental Information 1 [file peerj-09-11808-s001.docx]

| Number | Name | Distribution |
| --- | --- | --- |
| 1 | *Paeonia jishanensis* | Jishan and Yongji in Shanxi, China |
| 2 | *Paeonia ludlowii* | Linzhi and Milin in Tibet, China |
| 3 | *Paeonia delavayi* | Northwest Yunnan, Southwest Sichuan, China |
| 4 | *Paeonia qiui* | Shennongjia in Hubei, Xunyang in Shaanxi, China |
| 5 | *Paeonia decomposita* | Northwest Sichuan, China |
| 6 | *Paeonia ostii* | Gansu, Anhui, Shanxi and Henan, China |
| 7 | *Paeonia rotundiloba* | Western Sichuan |
| 8 | *Paeonia cathayana* | Henan Province, China |
| 9 | *Paeonia rockii* | Northern Sichuan, southern Gansu, China |

Table S1 **The nine species of tree peony.**
