# Supplementary material for: Single molecule, full-length transcript sequencing provides insight into the TPS gene family in Paeonia ostii
Source: PeerJ. 2021 Jul 15;9:e11808. doi: 10.7717/peerj.11808 (PMC8286706; doi:10.7717/peerj.11808)
Supplement: Supplemental Information 2 [file peerj-09-11808-s002.docx]

Table S2 **Primers used for reverse transcription and qRT-PCR.**

| **Name** | **Primer (5'-3')** |
| --- | --- |
| VN Primer | 5phos/ ACTTGCCTGTCGCTCTATCTTCTTTTTTTTTTTTTTTTTTTTVN |
| Strand-Switching Primer | TTTCTGTTGGTGCTGATATTGCTmGmGmG |

V=A, C, or G, N =A, C, G, or T

| **Gene name** | **Forward primer (5'-3')** | **Reverse primer (5'-3')** |
| --- | --- | --- |
| *PoTPS1* | ACCCGAGTTGGCGTGACAAA | TCATGCACCTGGCTTGTGAGT |
| *PoTPS3* | AGCACAGCTCCGGACCAAAC | TCCCTGGATTGTCGGCTGGT |
| *PoTPS4* | GGTTGGCAGTGTACGCATGG | TCGCTTCCGTCCAACAGCAC |
| *PoTPS5* | TGTTGGCCGGAAACCAAGCA | GACCCAGAAGCTTGGGAAGCA |
| *PoTPS6* | GTGCCCTCGAGATGGCTGAA | ACCTTCGTCGCGCATGATCT |
| *PoTPS7* | AGCACAGCTCCGGACCAAAC | TCCCTGGATTGTCGGCTGGT |
| *PoTPS8* | AGCTGCAGGGAACTTGGCA | GCGCATTTGTGCTTATGCAACG |
| *PoTPS9* | GTCGAAGTCAAGCCACAGGGA | GGTTTCCCGCCGTGGATCAT |
| *PoTPS10* | GGATTGGCTGCGGAGAAGGT | GGGTTCAGGCAGTGTTGGACT |
| *PoTPS11* | GCAATTGCGGCATGAGAAGCA | GCTCGCTCCAAATCCTGCAC |
| *ubiquitin* | GACCTATACCAAGCCGAAG | CGTTCCAGCACCACAATC |
