# Supplementary material for: Single molecule, full-length transcript sequencing provides insight into the TPS gene family in Paeonia ostii
Source: PeerJ. 2021 Jul 15;9:e11808. doi: 10.7717/peerj.11808 (PMC8286706; doi:10.7717/peerj.11808)
Supplement: Supplemental Information 3 [file peerj-09-11808-s003.docx]

Table S3 **Summary of ROIs from PacBio SMRT.**

| **Size(K)** | **Reads of insert** | **Read bases of insert** | **Mean read length of insert** | **Mean read quality of insert** | **Mean number of passes** |
| --- | --- | --- | --- | --- | --- |
| 1-2 | 78,236 | 209,879,051 | 2,682 | 0.93 | 10 |
| 2-3 | 86,376 | 158,740,370 | 1,837 | 0.93 | 15 |
| 3-6 | 66,124 | 249,613,899 | 3,774 | 0.91 | 7 |
| All | 230,736 | 618,233,320 | 2764,33 | 0.92 | 10.67 |
