# Supplementary material for: Single molecule, full-length transcript sequencing provides insight into the TPS gene family in Paeonia ostii
Source: PeerJ. 2021 Jul 15;9:e11808. doi: 10.7717/peerj.11808 (PMC8286706; doi:10.7717/peerj.11808)
Supplement: Supplemental Information 6 [file peerj-09-11808-s006.docx]

Table S4 **Summary of consensus sequence from PacBio single-molecule long-read sequencing.**

| **Size(kb)** | **Number of consensus isoforms** | **Average consensus isoforms read length** | **Number of polished high-quality isoforms** | **Number of polished low-quality**  **isoforms** | **Percent of polished high-quality isoforms（%）** |
| --- | --- | --- | --- | --- | --- |
| 0-1 | 1,925 | 912 | 1,763 | 162 | 91.58 |
| 1-2 | 16,305 | 1,399 | 14,581 | 1,724 | 89.43 |
| 2-3 | 13,604 | 2,346 | 11,676 | 1,928 | 85.83 |
| 3-6 | 12,638 | 3,665 | 8,730 | 3,908 | 69.08 |
| ＞6 | 534 | 9,297 | 17 | 517 | 3.18 |
| All | 45,006 | 17,619 | 36,767 | 8,239 | 67.82 |
