# Supplementary material for: Single molecule, full-length transcript sequencing provides insight into the TPS gene family in Paeonia ostii
Source: PeerJ. 2021 Jul 15;9:e11808. doi: 10.7717/peerj.11808 (PMC8286706; doi:10.7717/peerj.11808)
Supplement: Supplemental Information 7 [file peerj-09-11808-s007.docx]

Table S6 **Annotated databases New Isoform Number.**

| **Annotated databases** | **Isoform number** |
| --- | --- |
| COG | 12,892 |
| GO | 20,052 |
| KEGG | 13,487 |
| KOG | 18,842 |
| Pfam | 33,856 |
| Swiss-Prot | 21,929 |
| eggNOG | 28,606 |
| nr | 28,790 |
| All annotated | 28,850 |
