# Supplementary figures and images for: Single molecule, full-length transcript sequencing provides insight into the TPS gene family in Paeonia ostii
Source: PeerJ. 2021 Jul 15;9:e11808. doi: 10.7717/peerj.11808 (PMC8286706; doi:10.7717/peerj.11808)

melting curves:


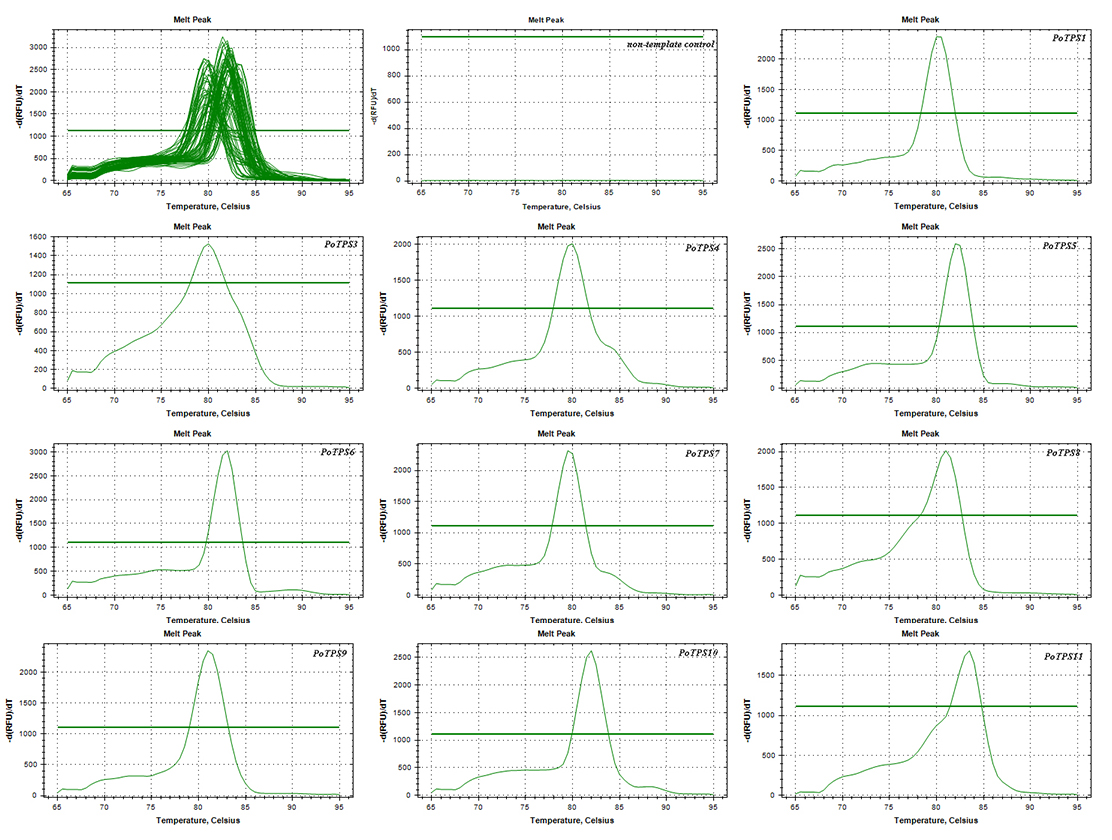

Supplement: Supplemental Information 8 [file peerj-09-11808-s008.docx]

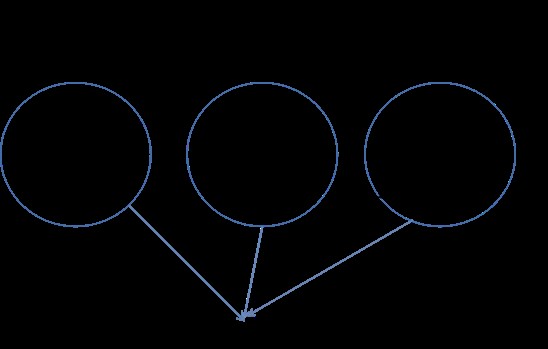

Supplement: Supplemental Information 11 [file peerj-09-11808-s011.jpg]
