# Supplementary material for: Single molecule, full-length transcript sequencing provides insight into the TPS gene family in Paeonia ostii
Source: PeerJ. 2021 Jul 15;9:e11808. doi: 10.7717/peerj.11808 (PMC8286706; doi:10.7717/peerj.11808)
Supplement: Supplemental Information 9 [file peerj-09-11808-s009.docx]

Table S5 **Prediction of SSRs out of our transcript datasets.**

| **Item** | **Numbers** |
| --- | --- |
| Total number of sequences examined | 30086 |
| Total size of examined sequences (bp) | 65294680 |
| Total number of identified SSRs | 13632 |
| Number of SSR containing sequences | 9789 |
| Number of sequences containing more than 1 SSR | 2639 |
| Number of SSRs present in compound formation | 1277 |
| Mono nucleotide | 9534 |
| Di nucleotide | 2052 |
| Tri nucleotide | 1814 |
| Tetra nucleotide | 59 |
| Penta nucleotide | 26 |
| Hexa nucleotide | 147 |
