# Supplementary material for: Single molecule, full-length transcript sequencing provides insight into the TPS gene family in Paeonia ostii
Source: PeerJ. 2021 Jul 15;9:e11808. doi: 10.7717/peerj.11808 (PMC8286706; doi:10.7717/peerj.11808)
Supplement: Supplemental Information 10 [file peerj-09-11808-s010.docx]

Table S7 **The percentage of similarity between these TPS family members and *AtTPS1-11 in Arabidopsis*.**

| Similarity（%） | *PoTPS1* | *PoTPS3* | *PoTPS4* | *PoTPS5* | *PoTPS6* | *PoTPS7* | *PoTPS8* | *PoTPS9* | *PoTPS10* | *PoTPS11* |
| --- | --- | --- | --- | --- | --- | --- | --- | --- | --- | --- |
| *AtTPS1* | 86.15 | 78.01 | 77.72 | 33.05 | 34.95 | 31.43 | 33.55 | 25.36 | 33.10 | 29.14 |
| *AtTPS2* | 67.56 | 60.05 | 56.77 | 34.88 | 35.00 | 34.32 | 35.23 | 29.30 | 36.02 | 33.18 |
| *AtTPS3* | 64.50 | 64.77 | 11.96 | 36.02 | 37.99 | 34.91 | 33.07 | 12.35 | 35.90 | 8.92 |
| *AtTPS4* | 67.65 | 65.65 | 63.80 | 32.77 | 35.21 | 31.91 | 33.82 | 27.08 | 33.58 | 32.67 |
| *AtTPS5* | 36.04 | 32.93 | 32.02 | 79.98 | 75.19 | 65.50 | 65.18 | 58.51 | 64.41 | 63.08 |
| *AtTPS6* | 35.44 | 33.66 | 31.64 | 74.17 | 83.83 | 67.30 | 63.94 | 63.07 | 62.60 | 62.87 |
| *AtTPS7* | 34.85 | 31.88 | 30.94 | 65.88 | 66.47 | 75.32 | 61.52 | 60.33 | 60.43 | 59.79 |
| *AtTPS8* | 34.54 | 31.65 | 29.90 | 62.75 | 64.36 | 61.01 | 71.45 | 70.89 | 70.76 | 69.43 |
| *AtTPS9* | 34.40 | 31.82 | 28.77 | 64.56 | 64.33 | 61.66 | 71.84 | 72.26 | 70.92 | 69.62 |
| *AtTPS10* | 35.16 | 31.57 | 28.71 | 61.71 | 62.24 | 60.12 | 71.13 | 71.56 | 70.95 | 68.99 |
| *AtTPS11* | 35.00 | 31.45 | 29.04 | 58.57 | 58.25 | 57.58 | 57.82 | 60.28 | 58.85 | 59.83 |
